# Differential gene expression in ripe mango fruit (*Mangifera indica* L. cv. Azúcar) that favors the pathogenicity of the endophyte *Colletotrichum tropicale*

**DOI:** 10.3389/ffunb.2025.1699983

**Published:** 2025-11-06

**Authors:** Andrés F. Quintero-Mercado, Sebastián Rojas, Yordan J. Romero-Contreras, Luis F. Lozano, Mario Serrano, Celsa García

**Affiliations:** 1 Facultad de Ciencias Agrarias, Departamento de Agronomía, Universidad Nacional de Colombia, Bogotá, Colombia; 2 Facultad de Ingeniería, Programa de Ingeniería Agronómica, Universidad del Magdalena, Santa Marta, Colombia; 3 Facultad de Ciencias, Departamento de Biología, Universidad Nacional de Colombia, Bogotá, Colombia; 4 Centro de Ciencias Genómicas, Universidad Nacional Autónoma de México, Cuernavaca, Morelos, Mexico

**Keywords:** *Colletotrichum tropicale*, genome-wide expression analysis, *Mangifera indica* L. cv. Azúcar, oxidative burst, oxidative stress, MAPK, quiescence

## Abstract

*Colletotrichum tropicale* is an endophyte that has been reported as a pathogen in ripe mango fruits (Mangifera indica L. cv. Azúcar) in Magdalena, Colombia, causing anthracnose. However, gene expression in the host that promotes its lifestyle transition remains unknown. This study aimed to analyze gene expression during the interaction between ripe mango fruit cv. Azúcar and *C. tropicale* to identify differentially expressed host genes that facilitate the pathogen’s infection process. RNA sequencing (RNA-seq) analysis was conducted at 0 and 12 h post inoculation (hpi), including *de novo* assembly and bioinformatic functional annotation using Gene Ontology (GO) and Kyoto Encyclopedia of Genes and Genomes (KEGG). A total of 5,435 differentially expressed genes (DEGs) were identified in the interaction, of which only 421 DEGs were detected in ripe mango fruits. Among these, 379 were upregulated and 42 were downregulated (T0 vs. T12 hpi). GO functional annotation of downregulated fruit genes revealed that the molecular functions affected at 12 hpi were related to the plant’s defensive oxidative burst mediated by reactive oxygen species (ROS)—including NADPH oxidase activity, hydrogen peroxide formation, and the action of peroxidases and oxidoreductase enzymes—whereas upregulated genes were associated with stress response, defense, transferase activity, and kinase activity. KEGG analysis identified pathways related to mitogen-activated protein kinase (MAPK) signaling, pathogen-associated molecular pattern (PAMP)-triggered immunity, and phenylalanine metabolism. In conclusion, ripe mango fruit cv. Azúcar activates a defense response against *C. tropicale* at 12 hpi that does not overcome the pathogen’s initial quiescent phase but instead facilitates conditions for its establishment by suppressing oxidative burst pathways, which may later contribute to oxidative stress during the necrotrophic phase.

## 1 Introduction

Over the past three decades, mango (*Mangifera indica* L.) cultivation has expanded in production area and increased its commercial supply to new international markets. This fruit is of great importance due to its high nutritional value, as it contains macronutrients such as carbohydrates, lipids, fatty acids, and amino acids, as well as micronutrients including vitamins and minerals (Ledesma and Campbell, 2019; Maldonado-Celis et al., 2019). According to Arcila Cardona et al. (2022), the mango cv. Azúcar has been naturalized in Colombia, where it is grown in warm and dry areas ranging from 11 to 556 m above sea level (m.a.s.l.), mainly in regions such as Cesar, Córdoba, Cundinamarca, Huila, Magdalena, and Tolima (García Lozano et al., 2010). Among these, Magdalena contributed 20% to the national mango production according to the Ministerio de Agricultura y Desarrollo Rural, Colombia (2025).

The production system of mango cv. Azúcar is the fourth most economically important crop in the region of Magdalena (Colombia), after banana, African oil palm, and coffee (Salcedo, 2018). It is affected by anthracnose, a disease caused by the phytopathogenic fungus *Colletotrichum*, which is considered the most limiting factor in mango production across tropical and subtropical regions (Arauz, 2000; de Souza et al., 2013; Kamle, 2013; Ploetz, 2007). In this region, the disease can cause yield losses ranging from 20% to 50% both in the field and postharvest (Páez, 2003), as well as severe damage that reduces the productivity and competitiveness of various agroecosystems and plants in general, with losses reaching up to 100% (Crouch et al., 2014; da Silva et al., 2020; De Silva et al., 2017; Vieira et al., 2014). Regarding the endophytism of *Colletotrichum*, few reports confirm that this fungus can undergo a phase shift to a pathogenic lifestyle in mango. This has been evidenced in studies by Quintero-Mercado et al. (2019) and Páez-Redondo et al. (2024), in which *C. tropicale* was obtained as an endophyte from asymptomatic leaves and fruits of the cv. Azúcar, respectively, and when inoculated onto detached fruits, necrotic lesions characteristic of anthracnose symptoms developed. Similarly, Vieira et al. (2014) reported that endophytic isolates from mango cv. Tommy Atkins, including *C. tropicale*, *C. asianum*, *C. endomangiferae*, *C. cliviae*, *C. dianesei*, *C. fructicola*, and *C. karstii*, exhibited significant virulence in pathogenicity assays on detached fruits. For the breakdown of a mutualistic interaction between the fungus and the plant to occur, predisposing conditions must be present—provided by the host (e.g., biochemical changes), by the pathogen’s virulence, or by favorable environmental conditions in the ecosystem (De Silva et al., 2017; Delaye et al., 2013). The ability of this fungal microorganism to remain asymptomatic within plant tissue poses a phytosanitary risk due to the hidden production of infective propagules or initial inoculum (Freeman et al., 2001).

According to Kogel et al. (2006), endophytes must overcome the plant’s nonspecific defense responses, achieving successful penetration of the cell wall through the reprogramming of invaded cells, which leads to adaptation of the infective structures formed. This process preserves host integrity and ensures a long-lasting interaction, or a “friendly” infection, which in some cases is mediated by recognition through plant transmembrane kinase receptors. In this regard, the host must recognize conserved molecules of certain microorganisms, known as microbe-associated molecular patterns (MAMPs) or pathogen-associated molecular patterns (PAMPs) (Khare et al., 2018). According to Sánchez-Vallet et al. (2015), endophytes also develop defense mechanisms against the plant’s immune response.

Pathogenic symbioses are characterized by the production of enzymes that degrade the plant cell wall, in which damage-associated molecular pattern (DAMP) receptors perceive cellular debris, adenosine triphosphate (ATP), and carbohydrates to initiate an immune signaling response by the host. In contrast, mutualistic symbioses do not cause apparent damage to the integrity of the plant cell, and DAMP receptors are possibly silenced, repressing the plant’s immune signaling pathways (Plett and Martin, 2018).

In the case of a lifestyle shift from mutualistic symbiosis to pathogenesis, it can be stated that the microorganism is capable of activating mitogen-activated protein kinase (MAPK) pathways and the enzymatic complex NADPH oxidase that produces reactive oxygen species (ROS), possibly through the secretion of apoplastic as well as cytoplasmic effectors, with the purpose of damaging the cell wall (Eaton et al., 2011).

The use of omics tools, such as transcriptomics through RNA sequencing (RNA-seq), has not been explored to understand the endophyte–pathogen relationship of *Colletotrichum* in mango, particularly in cv. Azúcar from the region of Magdalena, Colombia. Such studies would allow the establishment of transcriptional profiles to help elucidate the nature of mutualism/endophytism and the host immune response (Dinkins et al., 2017; Eaton et al., 2011; Kaul et al., 2016), generating new knowledge of the genetic factors influencing the variation in lifestyles of *Colletotrichum* in organs such as ripe fruits. This would lead to the development of more effective control strategies against anthracnose and strengthen the agro-industrial competitiveness of mango cv. Azúcar. In this study, the differential gene expression of ripe mango fruit cv. Azúcar that favors the pathogenicity of the endophyte *C. tropicale* was analyzed.

## 2 Materials and methods

### 2.1 Disinfection of ripe mango fruits cv. Azúcar for inoculation tests

According to the BBCH scale proposed by Rajan et al. (2011), ripe fruits in phenological stage 801, detached and completely healthy, were used. Disinfection was carried out following the methodology proposed by Quintero-Mercado et al. (2019). Under laminar flow chamber conditions, fruits were immersed, in the following order, in sterile distilled water for 2 min, in 1% sodium hypochlorite for 1 min, and again in sterile distilled water for 2 min. Subsequently, they were sprayed with 70% ethanol for 1 min, and the excess was removed using sterile distilled water. Once disinfected, the fruits were left to dry completely on sterile Kraft paper for 45 min.

### 2.2 Preparation of the inoculum and inoculation of *C. tropicale*

The fungal inoculum was prepared from pure cultures with 20 days of growth on potato dextrose agar (PDA) medium supplemented with streptomycin sulfate (200 mg/L), under conditions of 26°C, a 12 h light/12 h dark photoperiod, and 80% relative humidity. The endophytic isolate was obtained from asymptomatic leaves of mango cv. Azúcar. Its pathogenicity was confirmed, and it was identified as *Colletotrichum tropicale* (GenBank^®^ OR563797.1). In a laminar flow chamber, 20 mL of sterile distilled water were added to each culture, and the surface was scraped with a sterile spatula to collect the entire fungal content while avoiding contamination with the culture medium. The suspension was gently stirred and transferred to a sterile glass beaker, then macerated with a spatula and vortexed for 3 min. It was filtered through sterile medical gauze and collected in another aseptic glass beaker. The fungal suspension was adjusted to a concentration of 1 × 10^6^ conidia/mL.

Using the drop-deposition inoculation technique, the inoculum was applied to six equidistant points on the epicarp of each fruit, with a volume of 10 μL per point and a second application at each site for a total of 20 μL of suspension. Six fruits, considered one biological replicate, were used for each evaluation time point (0 and 12 h post inoculation [hpi]), following the histological study by Quintero-Mercado (2025), in which it was identified that at 12 hpi the fungus employs quiescence as a pathogenic strategy in ripe mango cv. Azúcar, behaving as a hemibiotroph. At 0 hpi, inoculation was performed with sterile distilled water, and one additional fruit was inoculated with the fungal suspension to monitor symptoms and characteristic signs of the disease. Once inoculated, the fruits were kept under high-humidity conditions inside hermetically sealed plastic containers at a temperature between 22 °C and 25°C and 80% relative humidity in an environmental test chamber (Sanyo, now PHCbi). For each evaluation time point (0 and 12 hpi), three biological replicates were used. In each tube, 2 mL of NucleoProtect RNA reagent (Macherey-Nagel) were added to preserve RNA integrity.

### 2.3 Transcriptomic analysis

The samples preserved in NucleoProtect RNA (Macherey-Nagel) were sent to the Beijing Genomics Institute (BGI, Hong Kong) for total RNA extraction, library construction, and sequencing using DNBSeq™ technology with a PE150 read length.

To assess sequence quality, FastQC (version 0.11.8) (https://www.bioinformatics.babraham.ac.uk/projects/fastqc/) was used, followed by trimming with Trim-Galore (version 0.6.10) (https://www.bioinformatics.babraham.ac.uk/projects/trim_galore/). *De novo* assembly was performed using Trinity (version 2.15.1) (Grabherr et al., 2011), pooling all biological replicates for each time point or condition (T0 hpi = 3; T12 hpi = 3). The resulting transcripts were translated into proteins using TRANSDECODER (version 5.7.1) (https://github.com/TransDecoder/TransDecoder). The quality of the *de novo* assembly was assessed by performing BLAST (version 1.15.0) (Altschul et al., 1990) against UniProt release 2024_02 (https://www.uniprot.org/), and by aligning the reads of each biological replicate to the assembled transcripts using BOWTIE2 (version 2.5.2) (Langmead and Salzberg, 2012). Expectation-maximization (RSEM) (version 1.3.3) (Li and Dewey, 2011) was used to estimate gene abundance in each replicate, and DESeq2 (version 3.19) (Love et al., 2014) was applied for differential expression analysis. A log2 fold change (FC) ≥ 2 or ≤ −2 was used as the threshold.

### 2.4 Functional gene annotation through Gene Ontology (GO) in ripe mango fruit cv. Azúcar

For functional annotation, a BLAST search (version 1.15.0) (Altschul et al., 1990) was performed using three databases: the complete and annotated genome of *Mangifera indica*, compared against the plant-specific Nr database. Proteins with at least 35% identity and 70% coverage were selected.

After obtaining statistically significant genes (p-value, FDR < 0.05), GO analysis was conducted using the Blast2GO^®^ program (https://www.blast2go.com) (Götz et al., 2008), included in the OmicsBox tool, for the upregulated and downregulated genes of ripe mango fruit to identify molecular functions, biological processes, and cellular components.

### 2.5 Kyoto Encyclopedia of Genes and Genomes analysis for differentially expressed genes in ripe mango fruit cv. Azúcar

An enrichment analysis of specific molecular pathways was performed using KAAS–KEGG (version 2.1) (Kanehisa, 2004) (https://www.genome.jp/tools/kaas/) for the differentially expressed genes of ripe mango fruit cv. Azúcar.

### 2.6 Validation of gene expression data in ripe mango fruit cv. Azúcar by qRT-PCR

Total RNA was extracted using the InviTrap^®^ Spin Plant RNA Mini Kit from 100 mg of inoculated tissue at different evaluation times (0, 12, and 24 h post inoculation [hpi]). RNA integrity was verified by electrophoresis on a 1.5% agarose gel. Samples were treated with DNase I (DNase I, Amplification Grade, Invitrogen™). For cDNA synthesis, the M-MLV Reverse Transcriptase Kit (Invitrogen™) was used.

Specific primers for each gene were designed with Primer3 (version 4.1.0) (Untergasser et al., 2012) and synthesized by Macrogen Inc. (Seoul, South Korea) (**Supplementary Table 1**). qRT-PCR was performed using the PowerTrack™ SYBR Green Master Mix Kit (Applied Biosystems™) and the QuantStudio™ 5 Real-Time PCR System (Applied Biosystems™, Thermo Fisher Scientific).

Amplification conditions were as follows: 50°C for 2 min and 95°C for 2 min, followed by 40 cycles of 95°C for 15 s and the optimal annealing temperature for each primer set for 60 s. Three biological replicates were used for each evaluation time and analyzed in technical triplicates. Results were normalized using the Pfaffl method and subsequently transformed to log_2_. Outliers were identified and removed using the IQR method.

Normality and homogeneity of variance were assessed for each gene and treatment. When assumptions were met, analysis of variance (ANOVA) followed by Tukey’s *post hoc* test was applied; otherwise, the Kruskal–Wallis test followed by Dunn’s test with Bonferroni correction for multiple comparisons was used. Actin was employed as the endogenous reference gene for expression normalization.

## 3 Results

### 3.1 Sequencing quality

The cleaning statistics of the reads, averaged across the three replicates for each evaluation time (0 and 12 hpi), yielded approximately 24 million reads for both cond_T0A and cond_T12A samples, with total read sizes of 7.1 Gb for T0A and 7.2 Gb for T12A. Quality parameters were Q20 > 96% and Q30 > 90% for all six samples, with a GC content of 44% (**Supplementary Table 2**).

### 3.2 Differential gene expression analysis of ripe mango cv. Azúcar fruit

**Figure 1:**
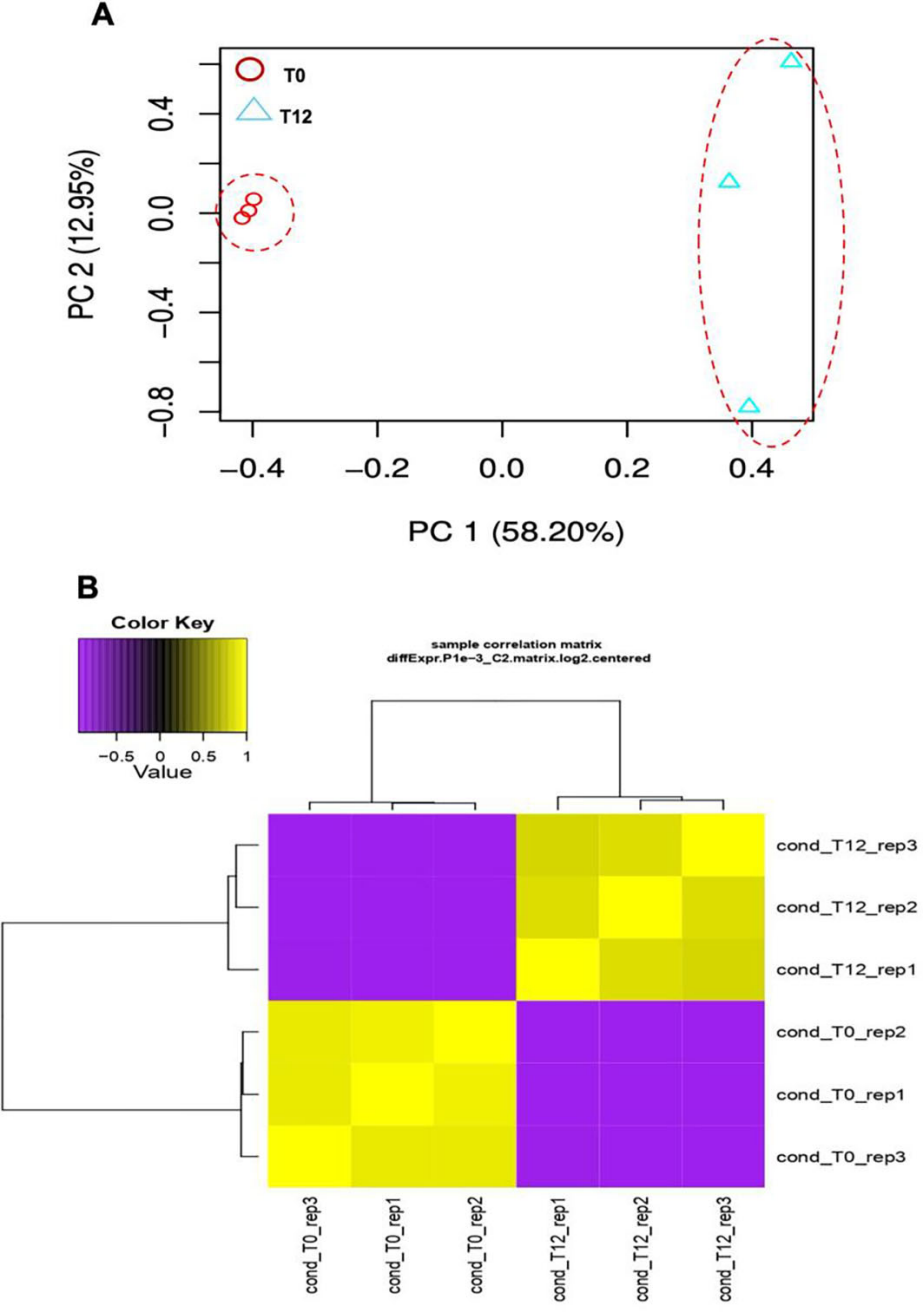
Transcriptomic analysis of the interaction between ripe mango fruit cv. Azúcar and *C*. *tropicale* under two evaluation time points (T0 vs. T12 h post inoculation). **(A)** Principal component analysis (PCA). Red dashed lines indicate separate clustering of each evaluation time point (T0 and T12 hpi). **(B)** Correlation heatmap of each evaluation time point (T0 and T12 hpi) across biological replicates (rep1, rep2, and rep3) (p < 0.05).

Differential expression analysis of ripe mango cv. Azúcar fruit inoculated with the endophyte *C. tropicale* was performed using principal component analysis (PCA), where PC1 accounted for 58.20% and PC2 for 12.95% of the total data variance (**Figure 1A**). The correlation heatmap (**Figure 1B**) showed consistent statistical behavior among the three biological replicates for each evaluation time (0 and 12 hpi).

**Figure 2:**
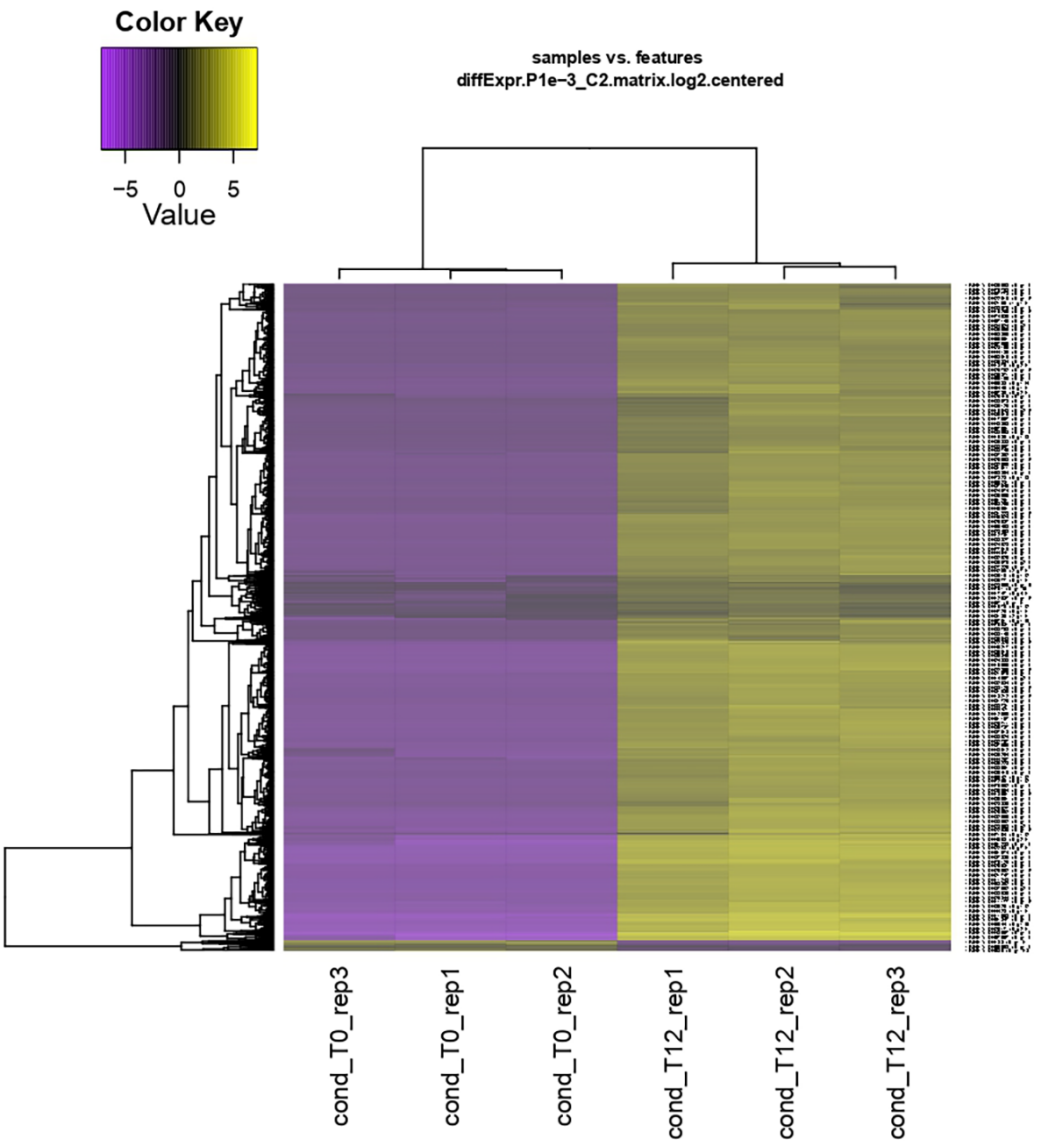
Hierarchical clustering and heatmap of differential expression analysis of the interaction between ripe mango fruit cv. Azúcar and *C. tropicale* at each evaluation time point (T0 and T12 hpi) across biological replicates (rep1, rep2, and rep3). Overexpressed genes are shown in yellow and repressed genes in purple. Log_2_ fold change (FC) ≥ 2 or ≤ −2 and p < 0.05.

From the clean reads, *de novo* assembly yielded 152,318 transcripts corresponding to 89,357 genes. A total of 5,435 differentially expressed genes (DEGs) were identified (T0 vs. T12 hpi), visualized in a heatmap with log_2_ fold change (FC) ≥ 2 or ≤ −2 and p < 0.05 (**Figure 2**).

### 3.3 Differentially expressed genes in ripe mango cv. Azúcar fruit

**Figure 3:**
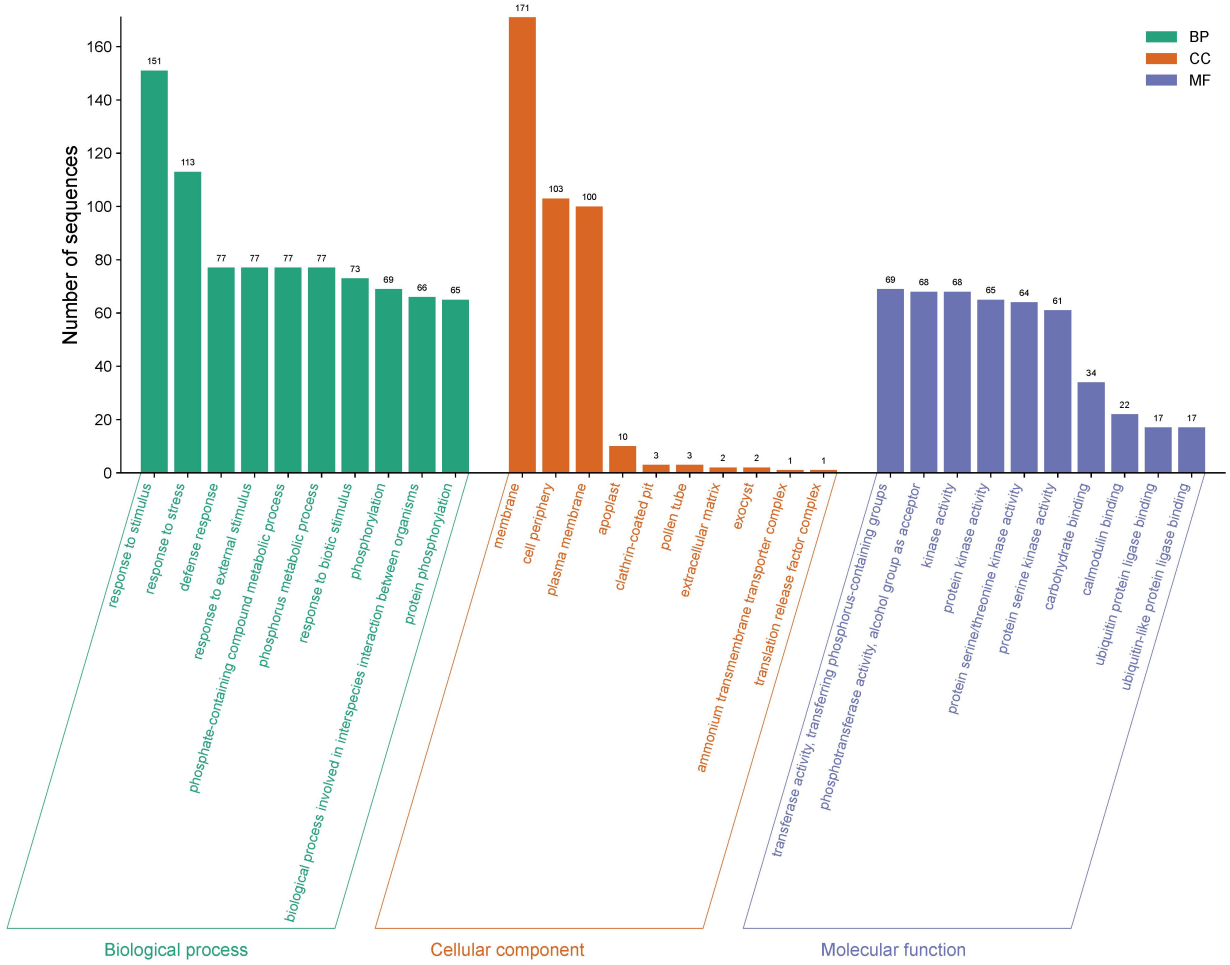
Gene Ontology (GO) terms for upregulated gene sequences in ripe mango fruit cv. Azúcar interacting with the endophyte *C. tropicale* at 12 hpi. Figure generated with SRPlot (Tang et al., 2023).

**Figure 4:**
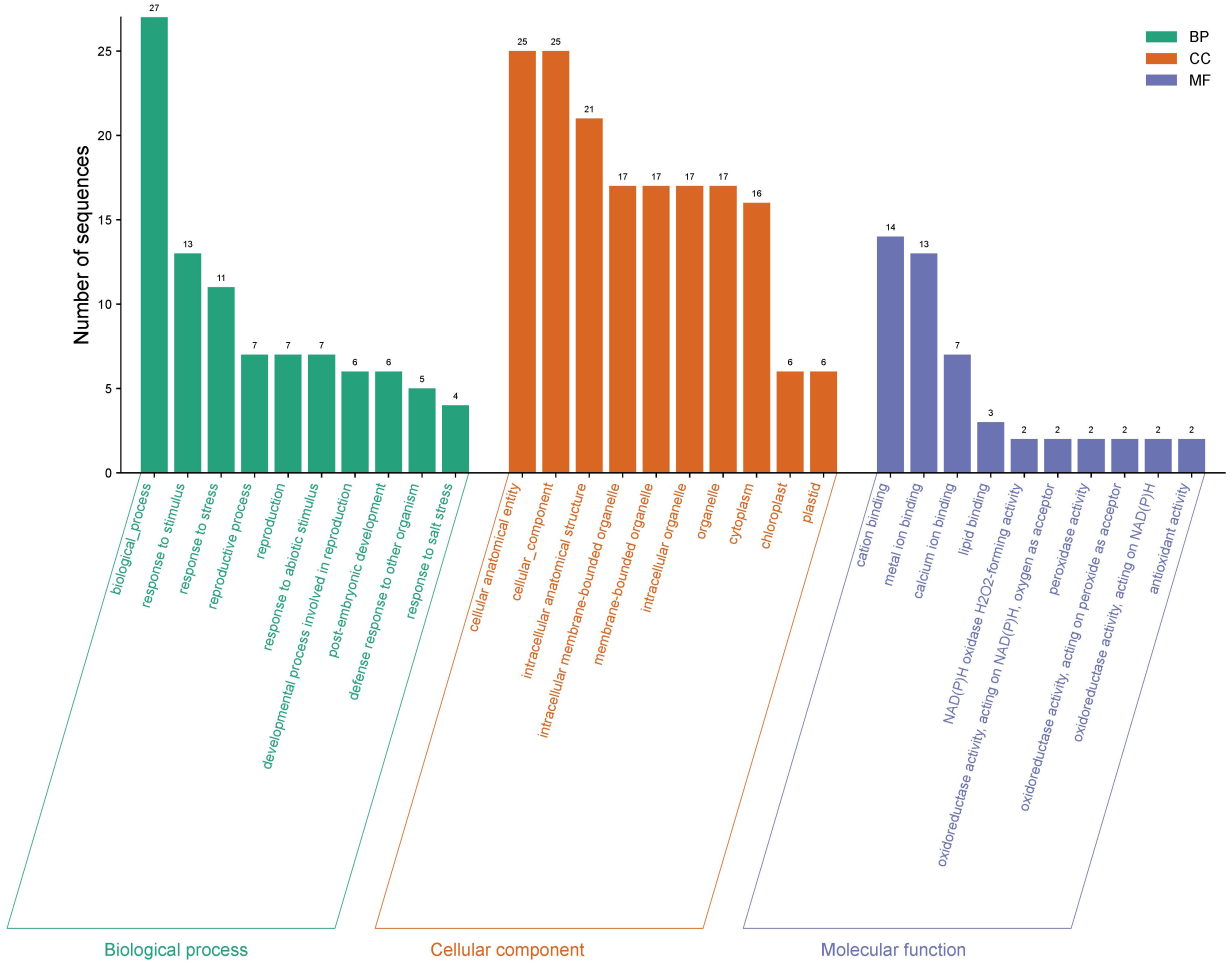
Gene Ontology (GO) terms for downregulated gene sequences in ripe mango fruit cv. Azúcar interacting with the endophyte *C. tropicale* at 12 hpi. Figure generated with SRPlot (Tang et al., 2023).

Of the 5,435 differentially expressed genes, only a total of 421 genes were identified in ripe mango fruit, with high significance against the Nr database for *Mangifera indica*, where 379 corresponded to upregulated genes and 42 to downregulated ones (T0 vs. T12 hpi) (**Supplementary Data Sheet 1**). Functional annotation by GO revealed that, for the upregulated genes, the largest number of sequences corresponding to gene subgroups were distributed in each category as follows: Biological Processes (response to stimulus: 151; response to stress: 113; defense response: 77), Cellular Components (membrane: 171; cell periphery: 103; plasma membrane: 100), and Molecular Functions (transferase activity: 69; phosphotransferase activity: 68; kinase activity: 68) (**Figure 3**). Regarding the downregulated genes, the highest number of sequences were grouped into the following subcategories: Biological Processes (biological process: 27; response to stimulus: 13; response to stress: 11), Cellular Components (cellular anatomical entity: 25; cellular component: 25; intracellular anatomical structure: 21), and Molecular Functions (cation binding: 14; metal ion binding: 13; calcium ion binding: 7) (**Figure 4**) (**Supplementary Data Sheet 2**).

### 3.4 GO enrichment analysis of differentially expressed genes

**Figure 5:**
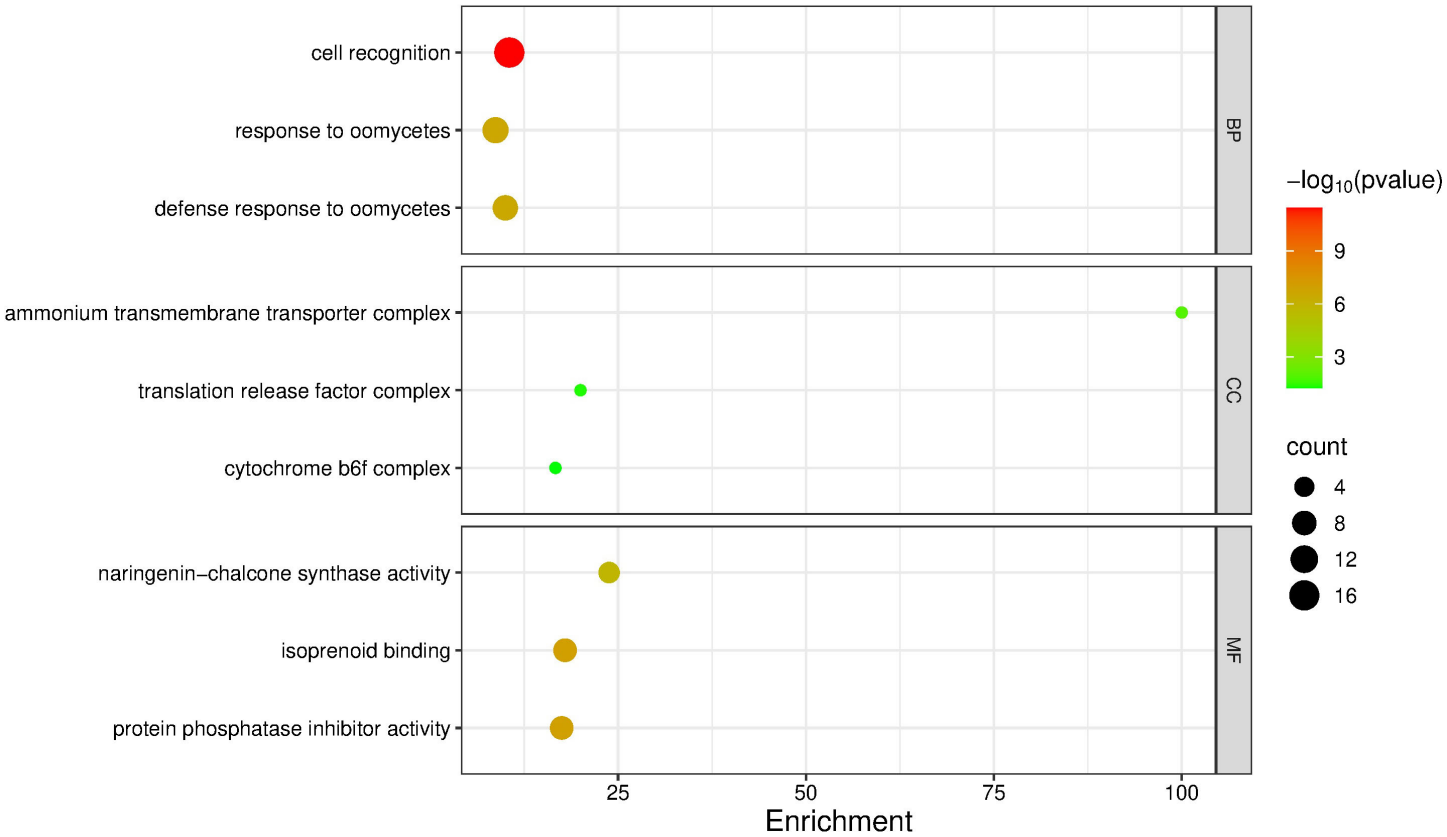
GO term enrichment analysis of upregulated genes in mango cv. Azúcar interacting with the endophyte *C. tropicale* at 12 hpi. Enrichment score (p < 0.05). Figure generated with SRPlot (Tang et al., 2023).

A GO enrichment analysis was performed, and the enrichment score was calculated by selecting the top three most enriched terms per GO category. For the upregulated genes, the most significantly enriched terms were: Biological Processes – cell recognition (enrichment score: 10.52; p = 3.60 × 10^-12^; 16 sequences), response to oomycetes (8.69; p = 2.70 × 10^-7^; 10 sequences), and defense response to oomycetes (10.00; p = 3.10 × 10^-7^; 9 sequences); Cellular Components – ammonium transmembrane transporter complex (100.0; p = 0.0115; 1 sequence), translation release factor complex (20.0; p = 0.0454; 1 sequence), and cytochrome b_6_f complex (16.6; p = 0.0564; 1 sequence); Molecular Functions – naringenin-chalcone synthase activity (23.08; p = 1.70 × 10^-6^; 5 sequences), isoprenoid binding (17.94; p = 9.80 × 10^-8^; 7 sequences), and protein phosphatase inhibitor activity (17.5; p = 1.20 × 10^-7^; 7 sequences) (**Figure 5**).

**Figure 6:**
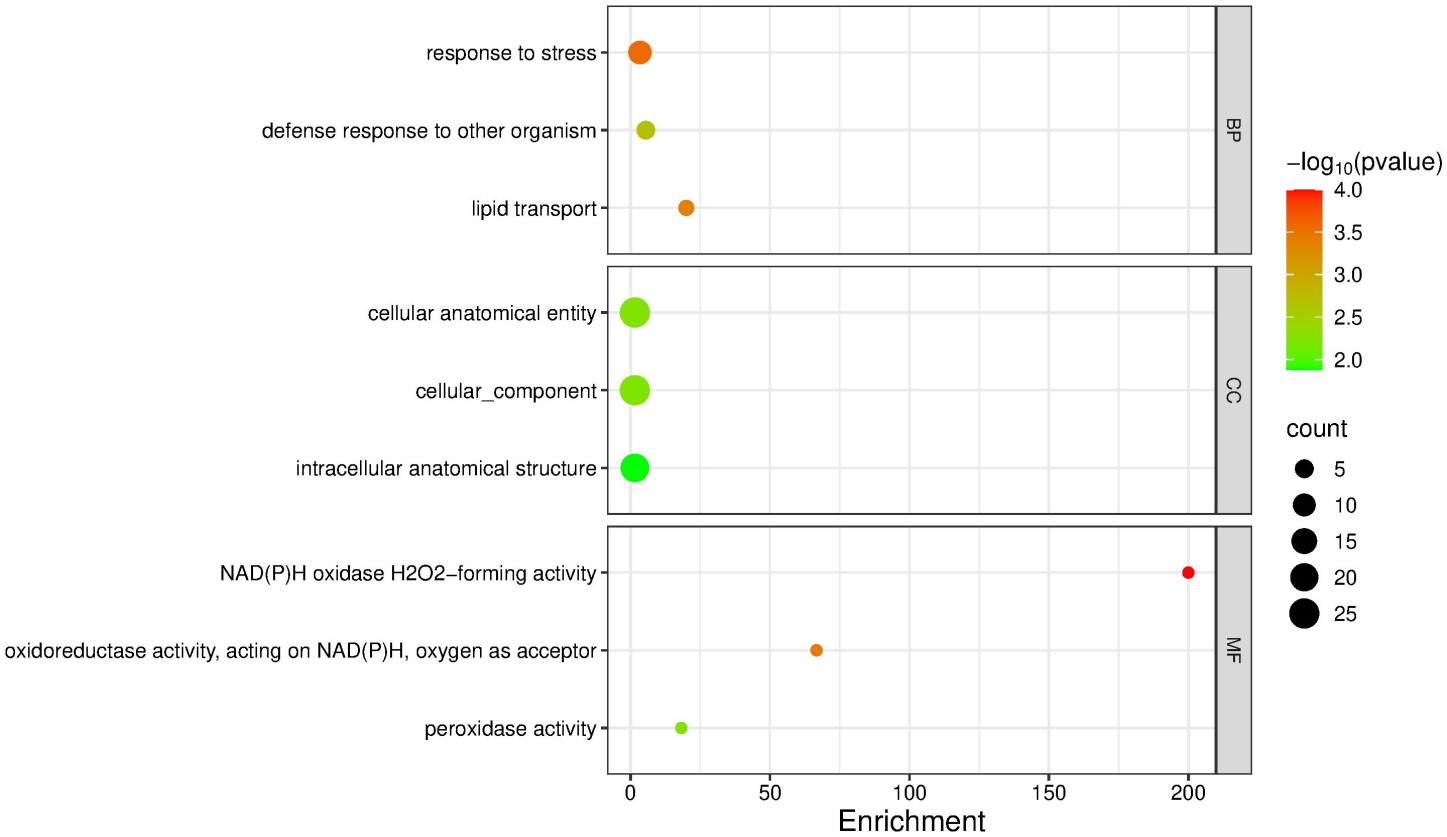
GO term enrichment analysis of downregulated genes in mango cv. Azúcar interacting with the endophyte *C. tropicale* at 12 hpi. Enrichment score (p < 0.05). Figure generated with SRPlot (Tang et al., 2023).

For the downregulated genes, the most enriched terms were: Biological Processes – response to stress (3.37; p = 2.70 × 10^-4^; 11 sequences), defense response to another organism (5.49; p = 0.0021; 5 sequences), and lipid transport (20.0; p = 4.30 × 10^-4^; 3 sequences); Cellular Components – cellular anatomical entity (1.54; p = 0.0056; 25 sequences), cellular component (1.54; p = 0.0059; 25 sequences), and intracellular anatomical structure (1.55; p = 0.0132; 21 sequences); Molecular Functions – NAD(P)H oxidase (H_2_O_2_-forming) activity (200.0; p = 0.0001; 2 sequences), oxidoreductase activity acting on NAD(P)H with oxygen as acceptor (66.6; p = 0.00037; 2 sequences), and peroxidase activity (18.1; p = 0.00567; 2 sequences) (**Figure 6**).

### 3.5 KEGG functional analysis of differentially expressed genes

**Figure 7:**
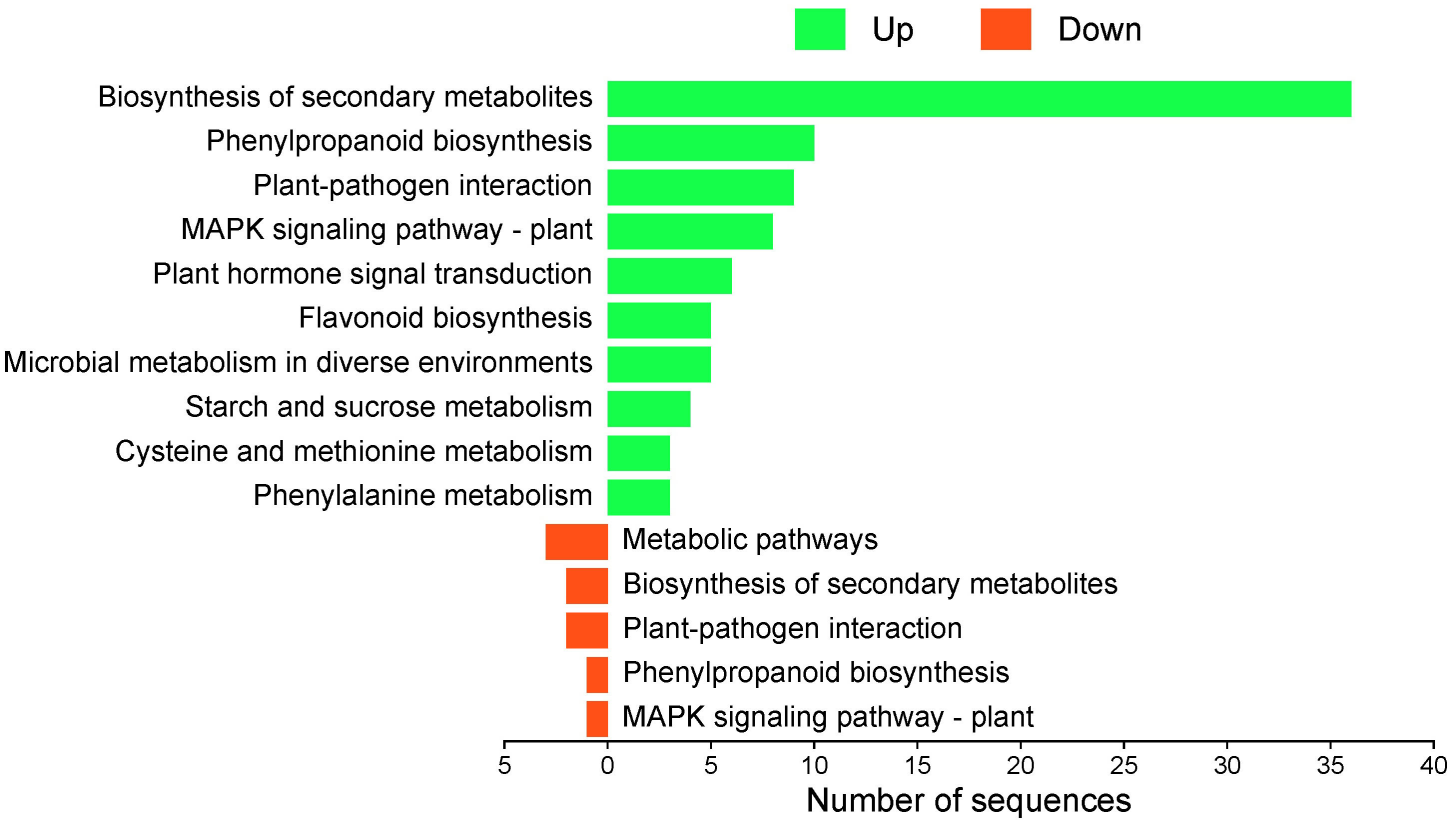
KEGG pathway enrichment analysis of differentially expressed genes in ripe mango fruit cv. Azúcar. Number of downregulated genes (red) and upregulated genes (green) at 12 hpi. Figure generated with SRPlot (Tang et al., 2023).

The KEGG functional analysis revealed that upregulated genes at 12 hpi were mainly associated with plant defense and secondary metabolism pathways. The most represented pathways included biosynthesis of secondary metabolites (36 genes), phenylpropanoid biosynthesis (10), plant–pathogen interaction (9), MAPK signaling pathway in plants (8), and plant hormone signal transduction (6). Conversely, downregulated genes were associated with general metabolic pathways (3), biosynthesis of secondary metabolites (2), plant–pathogen interaction (2), phenylpropanoid biosynthesis (1), and MAPK signaling pathway in plants (1) (**Figure 7**).

**Figure 8:**
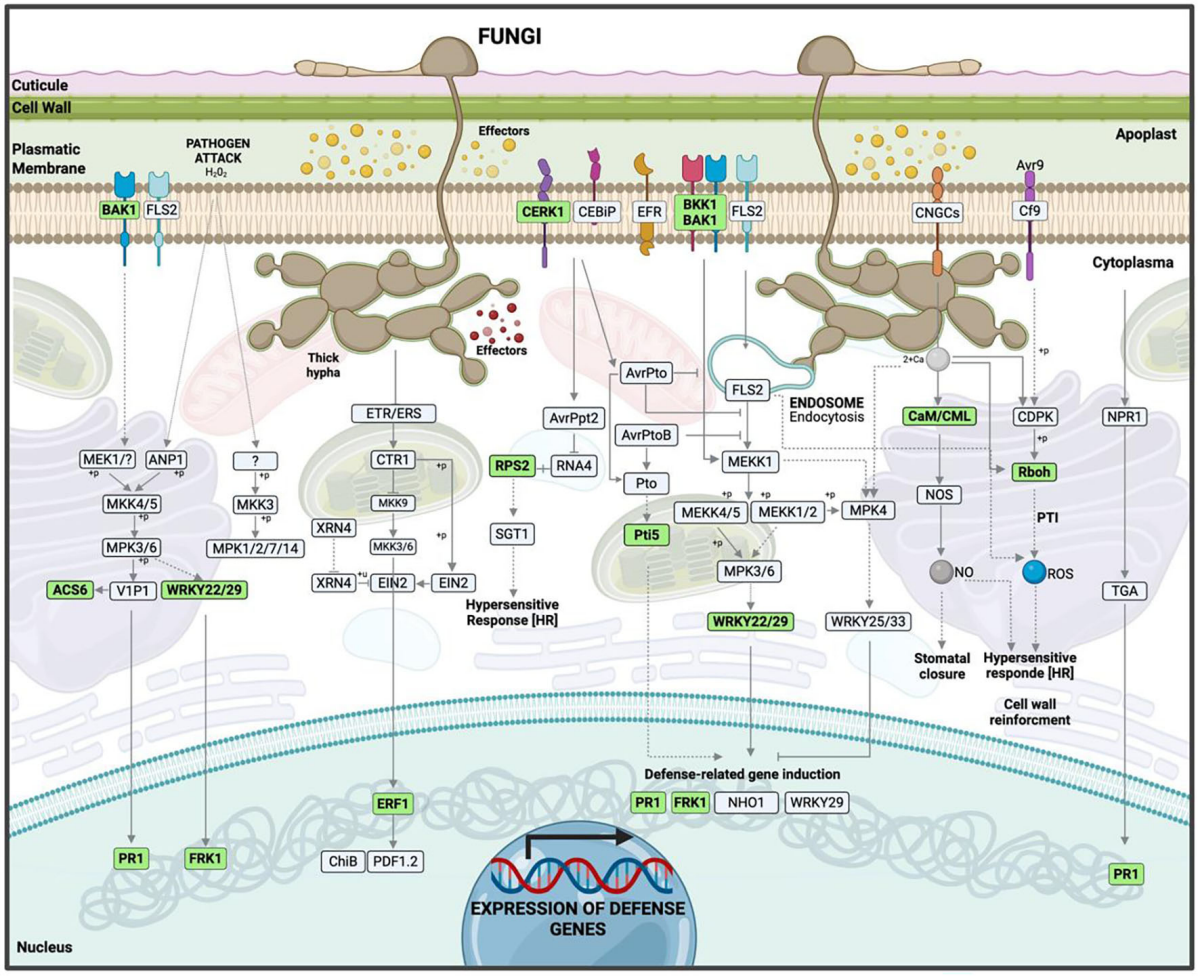
Hypothetical model of the interaction between ripe mango fruit cv. Azúcar and *C. tropicale* at 12 hpi, highlighting host upregulated pathways such as plant–pathogen interaction, plant hormone signal transduction, and MAPK signaling pathway – plant. Diagram created with BioRender.com.

Significant KEGG pathways were mapped according to highly enriched genes at 12 hpi. Among the downregulated genes, most were involved in PAMP-triggered immunity (PTI) within the KEGG map ko04626 (plant–pathogen interaction). The most relevant were *RbohC* (respiratory *burst oxidase homolog protein C*), a key component in reactive oxygen species (ROS) production, and *CaM/CML* (calmodulin/calmodulin-like proteins), fundamental in calcium-dependent signaling and nitric oxide (NO) synthesis (**Supplementary Figure 1**). Repression of these pathways compromises the hypersensitive response (HR) (**Figure 8**, **Supplementary Data 3**).

For upregulated genes, the same KEGG map (ko04626) revealed activation of both PAMP-triggered immunity (PTI) and effector-triggered immunity (ETI). Receptor and co-receptor genes such as *BAK1/BKK1* (brassinosteroid-insensitive 1-associated receptor kinase 1) and *CERK1* (chitin elicitor receptor kinase 1) were identified, indicating fungal pattern recognition to initiate early defense signaling.

Calcium and redox signaling pathways were also observed, involving *CaM/CML* (calmodulin) and *Rboh* (respiratory burst oxidase). Transcription factor and defense-related genes were detected, including *WRKY22/29*, *FRK1* (senescence-induced receptor-like serine/threonine-protein kinase), and *PR1* (pathogenesis-related protein 1), which contribute to plant immunity by regulating defense gene transcription, phytoalexin accumulation, and microRNA production.

Specific resistance genes such as *Pti5* (pathogenesis-related genes transcriptional activator PTI5) and *RPS2* (disease resistance protein RPS2) were identified, associated with ETI (**Supplementary Figure 2**, **Supplementary Data Sheet 3**).

The phenylalanine metabolism pathway (ko04075: plant hormone signal transduction) was also identified among the upregulated genes, with *PR1* activated, suggesting that *PR1* expression is induced through salicylic acid (SA) biosynthesis to trigger a defense response (**Supplementary Figure 3**).

**Figure 9:**
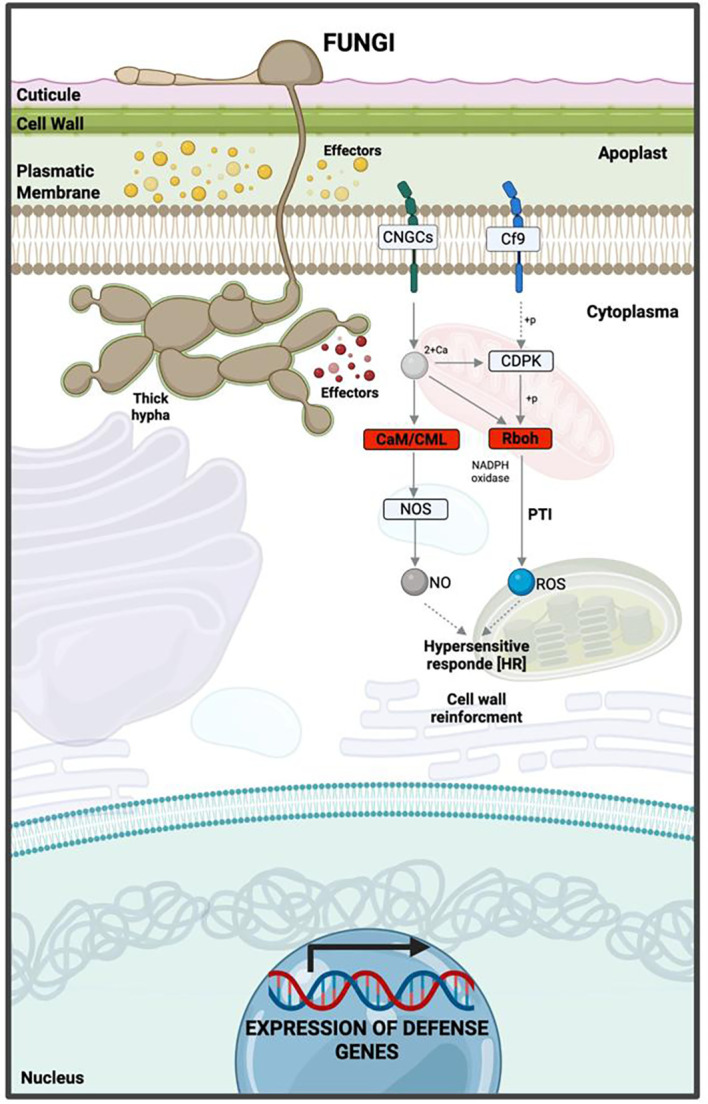
Hypothetical model of the interaction between ripe mango fruit cv. Azúcar and *C. tropicale* at 12 hpi, highlighting host downregulated pathways such as Rboh (respiratory burst oxidase homolog) and CaM/CML (calmodulin/calmodulin-like proteins). Diagram created with BioRender.com.

The MAPK signaling pathway (ko04016) revealed upregulated genes associated with pathogen perception and activation of the MAPK cascade (*BAK1, BRI1-associated kinase 1*); transcriptional responses through MAPK-activated factors (*WRKY22/29*); hormone biosynthesis such as ethylene (*ACS6, 1-aminocyclopropane-1-carboxylate synthase 6*; *ERF1*, ethylene response factor *1*); expression of defense genes (*FRK1, Flg22-induced receptor-like kinase 1*; *PR1*, pathogenesis-related *protein 1*); and activation of ROS production (*RbohD*, respiratory *burst oxidase homolog protein D*) (**Supplementary Figure 4**, **Figure 9**).

### 3.6 Validation of gene expression data in ripe mango cv. Azúcar by qRT-PCR

For qRT-PCR validation in ripe mango cv. Azúcar at 0, 12, and 24 hpi with *C. tropicale*, genes with the highest statistical significance (p < 0.05) were selected: one upregulated (*LOX1*: TRINITY_DN28581_c0_g1) and two downregulated (*THI4*: TRINITY_DN27837_c0_g1; *MT3*: TRINITY_DN25225_c1_g1).

**Figure 10:**
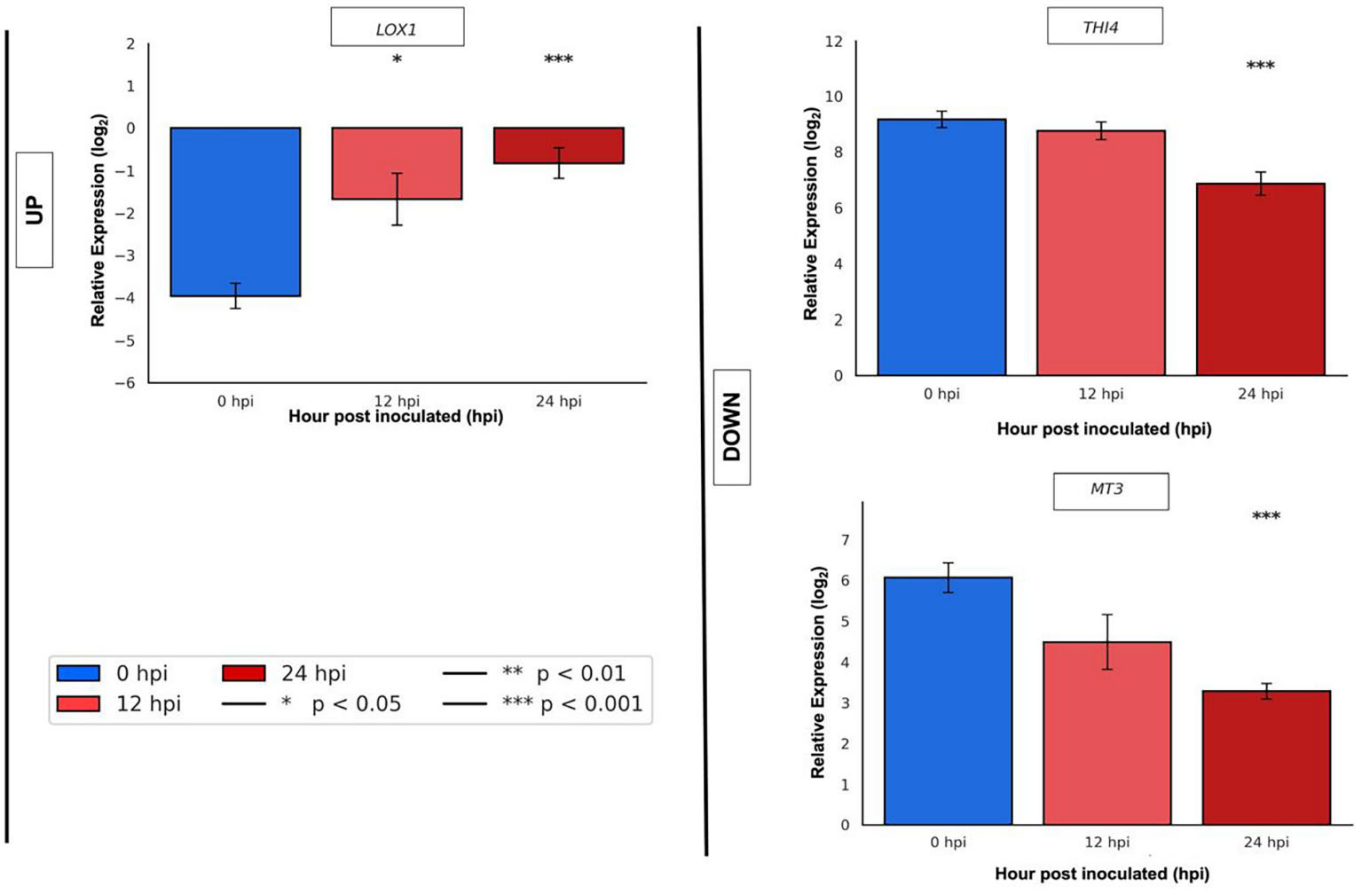
qRT-PCR validation of selected upregulated gene (*LOX1*: TRINITY_DN28581_c0_g1) and downregulated genes (*THI4*: TRINITY_DN27837_c0_g1; *MT3*: TRINITY_DN25225_c1_g1) in ripe mango fruit cv. Azúcar at 0, 12, and 24 hpi with the endophyte *C. tropicale*.

*LOX1* showed low expression at 0 hpi, which increased at 12 hpi (p < 0.05) and continued rising at 24 hpi (p < 0.001) (**Figure 10**). *THI4* decreased slightly at 12 hpi and more strongly at 24 hpi (p < 0.001). *MT3* displayed a progressive decrease beginning at 12 hpi and continued with marked significance (p < 0.001) at 24 hpi (**Figure 10**).

## 4 Discussion

The analysis of overexpressed genes in ripe mango fruit cv. Azúcar at 12 hpi reveals a significant activation of GO terms related to kinase activity, serine/threonine residue phosphorylation, calmodulin binding, and ubiquitin ligase activity. In other words, during the interaction with the endophyte *C. tropicale*, signaling pathways associated with pathogen perception, defense signal transduction, and protein regulation through the ubiquitin–proteasome system were activated. Regarding the role of protein kinases and receptors with carbohydrate-binding capacity, it is specified that their activation depends on pathogen-associated molecular patterns (PAMPs), leading to an initial defensive response. Likewise, the overexpressed genes associated with calmodulin and ubiquitin indicate their involvement in calcium-dependent signaling pathways and protein remodeling. This suggests that the ripe fruit perceives the presence of the fungus, triggering a robust defense response that does not ultimately result in the complete elimination of the pathogen.

The transcriptomic study conducted by Alkan et al. (2015) on the interaction between green/ripe tomato fruit and *C. gloeosporioides* reported host genes overexpressed during the quiescent phase of the fungus, with functions related to kinase activity, calmodulin binding, and ubiquitin ligases. Moreover, the pathogen was shown to alkalinize plant tissue to stimulate signaling pathways dependent on calcium, reactive oxygen species (ROS), and serine/threonine kinases.

For the repressed genes in ripe mango fruit cv. Azúcar, the GO results suggest that at 12 hpi, corresponding to the biotrophic stage of *C. tropicale*, the fungus impacts molecular functions associated with redox homeostasis, including NADPH oxidase involved in hydrogen peroxide production, peroxidases, antioxidant activities, and oxidoreductase enzymes. These functions are critical for the generation of ROS, which are essential for the activation of the host immune response, such as the hypersensitive response (HR).

Reactive oxygen species (ROS), including singlet oxygen (¹O_2_), superoxide anion (O_2_^-^), hydroxyl radical (OH•), nitric oxide (•NO), and hydrogen peroxide (H_2_O_2_) (Groß et al., 2013; Shetty et al., 2008; Wang et al., 2010), are released into the apoplast to establish the oxidative burst, which constitutes a plant defensive reaction in response to pathogen attack (Kámán-Tóth et al., 2019; Marino et al., 2012). For ROS generation, plant cells employ enzymatic activities related to class III peroxidases, NADPH oxidases, amine and polyamine oxidases (AOs and PAOs), respiratory burst oxidase homologs (RBOHs), glycolate oxidase, oxalate oxidase, xanthine oxidase, photosynthesis, photorespiration, and respiration (Baxter et al., 2014; Heller and Tudzynski, 2011; Kärkönen and Kuchitsu, 2015; Marino et al., 2012). Oxidative stress occurs when there is an imbalance between ROS production and antioxidant activity, which can lead to severe cellular damage and ultimately programmed cell death. Therefore, enzymes such as peroxidases, among others, are required to maintain system homeostasis through detoxification (dos Santos and Franco, 2023; Hasanuzzaman et al., 2017).

Macho and Zipfel (2015) reported that cytoplasmic effectors can silence RBOH enzymes and, consequently, inhibit ROS production. Similarly, Singh et al. (2016) suggested that these effectors can suppress RBOH-mediated pathways, whose function is to generate robust ROS production. Jwa and Hwang (2017) indicated that apoplastic effectors can eliminate ROS from the extracellular space to reduce pathogen damage and promote intracellular infection, through the silencing of PAMP recognition receptors (PRRs), which in turn inactivate NADPH oxidase-dependent RBOH pathways. Segal and Wilson (2018) reported that pathogens can eliminate host ROS to suppress its defensive response. Through RNA-seq studies, Alkan et al. (2015) identified that *Colletotrichum gloeosporioides*, to accomplish its lifestyle transition from quiescent to necrotrophic in green tomato fruit, can modulate the host redox environment by inducing alkalinization and delaying ROS activation. Studies conducted by Zhang et al. (2021) described that the hemibiotroph *C. orbiculare* produces the specific effector SIB1, which suppresses the oxidative burst or ROS production in *Nicotiana benthamiana*, with its highest expression observed at 12 hpi (during appressorium formation or the early infection stage). The oxidative burst in plants, as a defense strategy induced by the release of ROS and mediated by H_2_O_2_ during the hemibiotrophic stage of fungi, can be reduced at this stage and subsequently increase during the transition to the necrotrophic lifestyle. Eloy et al. (2015), in the cowpea (*Vigna unguiculata*)–*C. gloeosporioides* pathosystem, demonstrated that changes in H_2_O_2_ levels modulate fungal lifestyle transitions, with the necrotrophic stage being favored by the increase in ROS. The effectors *ChEC3, ChEC3a, ChEC5, ChEC6*, and *ChEC34* from *C. higginsianum* reported by Kleemann et al. (2012), as well as *CgDN3* from *C. orbiculare* described by Yoshino et al. (2012), were shown to be overexpressed and to suppress cell death in *N. benthamiana* leaves during the biotrophic phase. Gao et al. (2024) identified the *CfMsg5* gene of *C. fructicola* during its interaction with *Camellia oleifera* through RNA-seq analysis, which contributes to ROS detoxification, regulation of the MAPK cell wall integrity (CWI) pathway, and unfolded protein response (UPR), thereby promoting its pathogenicity.

In this context, the endophyte *C. tropicale*, which behaves as a hemibiotroph, can repress oxidative burst pathways associated with defense in ripe mango fruit cv. Azúcar, thereby facilitating its establishment during the quiescent or biotrophic phase. This repression could lead to oxidative stress and ROS overaccumulation, which may subsequently promote cell death and, consequently, the necrotrophic phase of *C. tropicale*, consistent with its hemibiotrophic lifestyle.

These results, concerning the overexpressed genes in mango cv. Azúcar, are consistent with the findings of Plett and Martin (2018), who reported that hemibiotrophs can activate plant defense responses through pathways related to PR proteins, ethylene, salicylic acid (SA), and ROS, leading to a hypersensitive response (**Figure 10**). In the case of ethylene, it has been described that Ethylene signaling increases during the quiescent phase, along with defense responses, and may even induce release from this same phase (Alkan et al., 2015). Hong et al. (2016), through RNA-seq studies on the mango fruit cv. Zill–*C. gloeosporioides* interaction, reported the role of overexpressed PR and ERF genes as positive regulators of host resistance against fungal infection. Our study reveals that ripe mango fruit cv. Azúcar is capable of overexpressing genes associated with ethylene and salicylic acid pathways.

Prusky et al. (2013) stated that ripe fruit reduces its antifungal defense against quiescent fungi, which induce high levels of ethylene and salicylic acid, thereby promoting programmed cell death. This is consistent with our study, which reveals that ripe mango fruit cv. Azúcar activates defense pathways mediated by ethylene and salicylic acid in response to infection by the endophyte *C. tropicale.*

*LOX* genes (linoleate:oxygen oxidoreductase; lipoxygenases) belong to the family of fatty acid or lipid substrate dioxygenases (Feussner and Wasternack, 2002*). In* plants, they are key enzymes that synthesize oxylipins, which act as signaling molecules during plant–pathogen interactions, leading to the production of *C_6_* volatiles and jasmonates (Viswanath et al., 2020). Studies by Peng et al. (1994) demonstrated that *LOX1* is induced early in rice as a response to infection by *Magnaporthe grisea*. Similarly, pepper leaves inoculated with *Xanthomonas campestris* and *Colletotrichum coccodes* showed high expression levels of the *CaLOX* gene, and in *Arabidopsis*, overexpression of the same gene conferred increased resistance to infection by *P. syringae*, *Hyaloperonospora arabidopsidis*, and *Alternaria brassicicola* (Hwang and Hwang, 2010). Hou et al. (2018) determined the role of the *DkLOX3* gene in *Arabidopsis*, where its overexpression enhanced resistance to *P. syringae* and *Botrytis cinerea*, as evidenced by greater ROS accumulation followed by cell death. Rustérucci et al. (1999) proposed that the synthesis of fatty acid hydroperoxides and volatile products with signaling roles act as precursors for LOX genes to mount pathogen defense. Likewise, these genes have been shown to confer antimicrobial activity (Croft et al., 1993; Weber et al., 1999). *LOX*-derived products also participate in hypersensitive response (HR) processes that enable the plant to contain pathogen infection and thereby establish a defense mechanism (Viswanath et al., 2020).

Joshi et al. (2020) reported that plants can use *THI4* (thiazole synthase), which leads to thiamine biosynthesis. Among the main functions of thiamine (vitamin B_1_) in plants under stress conditions, a prominent role is its ability to trigger ROS release and subsequently induce resistance (Li et al., 2016; Yin et al., 2022). Yang et al. (2024) demonstrated that the thiazole moiety of thiamine (THI2 = *TaTHI2*) plays an important role in plant immunity against *Chinese wheat mosaic virus* (CWMV), as it activates ROS production and enhances resistance in wheat.

Metallothioneins (MTs) are characterized as low molecular weight (6–7 kDa) metal-binding proteins with high cysteine content (~30%) (Li et al., 2023). In plants, MT genes such as MT3 are expressed in ripe fruits (Yang et al., 2015), and among their main functions is the regulation or neutralization of reactive oxygen species (ROS), which cause DNA damage (Akashi et al., 2004; Chubatsu and Meneghini, 1993; Miura et al., 1997; Wong et al., 2004). Dauch and Jabaji-Hare (2006) provided the first report of an MT gene (*MT3*) induced in *Abutilon theophrasti* plants in response to *Colletotrichum coccodes* infection, where the gene expression decreased 5 days after fungal inoculation, coinciding with the appearance of the first disease symptoms.

These results confirm that the endophyte *C. tropicale*, at early stages of its pathogenic process (hemibiotrophic with quiescence) at 12 hpi, is able to modulate defense response genes in ripe mango fruit cv. Azúcar—upregulating genes such as *LOX1*, which participates in the hypersensitive response (HR), and downregulating genes involved in primary metabolism and antioxidant homeostasis, such as *THI4* and *MT3*. This modulation could lead to oxidative stress that, in turn, favors the necrotrophic phase of the endophyte, as observed at 24 hpi, where the increased expression of *LOX1* may contribute to enhanced ROS release accompanied by cell death, while the decreased expression of *MT3* would reduce its capacity to scavenge ROS and to provide cellular protection.

## 5 Conclusions

Based on the RNA-seq study of the interaction between ripe mango fruit cv. Azúcar and the endophyte *C. tropicale*, 5,435 differentially expressed genes were identified from both the host and the fungus at 12 hpi. The main findings include: (1) repression of genes in ripe mango fruit associated with molecular functions involved in the plant’s defensive oxidative burst mediated by ROS (NADPH oxidase with hydrogen peroxide production, peroxidases, antioxidant activities, and oxidoreductase enzymes), possibly as an effect of colonization by the endophyte *C. tropicale* to establish its initial infective stage (quiescence) as a hemibiotroph; (2) validation of repressed genes in ripe fruit, *THI4* and *MT3*, with functions related to ROS release and ROS scavenging, respectively; (3) activation of signaling pathways in ripe fruit linked to pathogen perception, defense signal transduction, and protein regulation via the ubiquitin–proteasome system.

Altogether, these findings lead to the conclusion that ripe mango fruit cv. Azúcar initiates a defense response against the endophyte *C. tropicale* that does not fully overcome its initial pathogenic process at the cellular level through quiescence but rather creates favorable conditions for its establishment by suppressing oxidative burst pathways, which subsequently promote oxidative stress and facilitate the transition to its necrotrophic phase.

Thus, this study reveals the gene expression patterns that regulate the lifestyle switch of an endophyte such as *C. tropicale* in ripe mango fruit cv. Azúcar, based on its pathogenic strategy as a hemibiotroph with quiescence. The host transcriptional profile revealed in this study could be used to propose management strategies for anthracnose during the postharvest stage of ripe mango fruits cv. Azúcar, focusing on the external induction of defense responses and on the regulation of redox balance through antioxidants that counteract oxidative stress, thereby preventing the activation of the necrotrophic phase of the fungus.

## Data Availability

The RNA sequencing data have been deposited in the Sequence Read Archive (SRA) of the National Center for Biotechnology Information (NCBI), with accession number PRJNA1337713.

## Supplementary material

Supplementary Data Sheet 1 DEGs_Mango_cv_Azúcar_T0vsT12.

Supplementary Data Sheet 2 GO_Mango_cv_Azúcar_UP_DOWN.

Supplementary Data Sheet 3 KEGG_Mango_cv_Azúcar.
