# Supplementary material for: Differential gene expression in ripe mango fruit (Mangifera indica L. cv. Azúcar) that favors the pathogenicity of the endophyte Colletotrichum tropicale
Source: Front Fungal Biol. 2025 Nov 6;6:1699983. doi: 10.3389/ffunb.2025.1699983 (PMC12631287; doi:10.3389/ffunb.2025.1699983)
Supplement: Supplementary file 4 [file DataSheet1.docx]

Supplementary Material

# Supplementary Data

**Data file 1** DEGs_Mango_T0vsT12

**Data file 2** GO_Mango_UP_DOWN

**Data file 3** KEGG_Mango

# Supplementary Tables

**Supplementary Table 1.** Primers used for the validation of gene expression in ripe mango fruit cv. Azúcar by qRT-PCR.

| **Primer** | **Sequence** |
| --- | --- |
| **Reference gene** | |
| *Actina_F* | CATTGTGCTCAGTGGTGGTT |
| *Actina_R* | TTGGAGCAAGTGCAGTGATT |
| **Downregulated genes**  ID:TRINITY_DN25225_c1_g1 | |
| *MT3a_F* | AGTGTGAAGAAGGGAAGCGG |
| *MT3a_R* | TCGTGGTTCTCAGAAGTGGC |
| *MT3b_F* | GTGAAGAAGGGAAGCGGCTA |
| *MT3b_R* | CTCAGAAGTGGCAGGAACGT |
| ID: TRINITY_DN27837_c0_g1 | |
| *THI4a_F* | TTCAACCTCTTCACACGGGT |
| *THI4a_R* | AAACAGAGTGGGTGGTGTGG |
| *THI4b_F* | GGGCCATGCAAGATTGTGTG |
| *THI4b_R* | TGGAAGGAAACAGAGTGGGTG |
| **Upregulated genes**  ID: TRINITY_DN28581_c0_g1 | |
| *LOX1a_F* | TGGTCACTTGAAGATGGCAGA |
| *LOX1a_R* | CAAATACAGCTTCTAATGCAGGC |
| *LOX1b_F* | CAGGCTGCCACTTCTGATGA |
| *LOX1b_R* | ACAGCTTCTAATGCAGGCTCA |

**Supplementary Table 2.** Statistical analysis of sequencing data filtering (T0A and T12A hpi).

| **Sample** | **Number of clean reads** | **Read length**  **(Gb)** | **Q20**  **(%)** | **Q30**  **(%)** | **GC**  **(%)** |
| --- | --- | --- | --- | --- | --- |
| T0A_HKYR24020001_A | 23,991,947 | 7,1 | 96.86 | 90.83 | 44.32 |
| T0A_HKYR24020002_A | 24,245,310 | 7,2 | 96.90 | 91.13 | 43.89 |
| T0A_HKYR24020003_A | 24,117,073 | 7,2 | 96.77 | 90.87 | 43.95 |
| T12A_HKYR24020004_A | 24,244,621 | 7,2 | 96.63 | 90.49 | 44.56 |
| T12A_HKYR24020005_A | 24,145,243 | 7,2 | 96.79 | 90.71 | 44.62 |
| T12A_HKYR24020006_A | 24,317,186 | 7,2 | 96.63 | 90.34 | 44.84 |

# Supplementary Figures


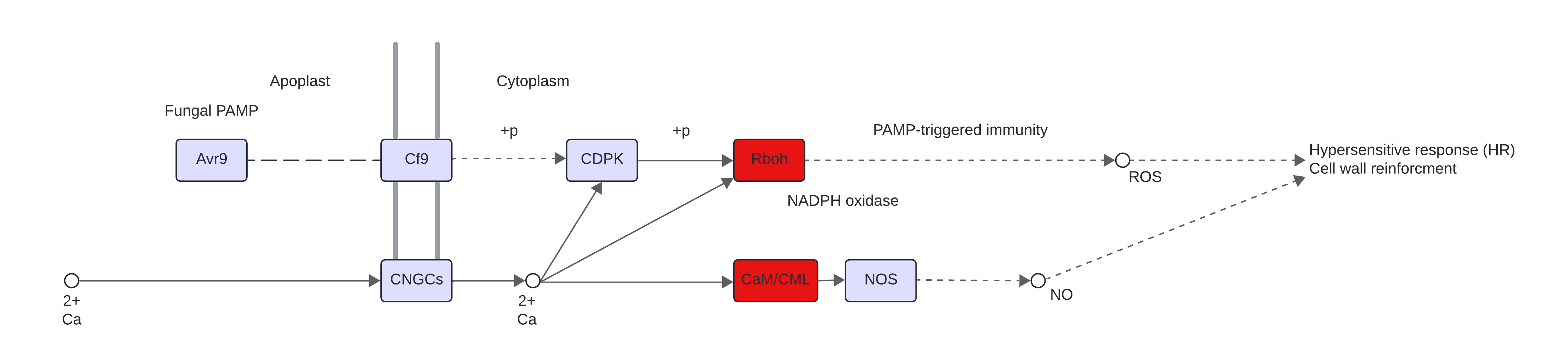


**Supplementary Figure 1.** KEGG map (ko04626: Plant–pathogen interaction) identified for downregulated genes in ripe mango fruit cv. Azúcar at 12 hpi. Genes *Rboh* and *CaM/CML* involved are highlighted in red. Figure created with Lucidchart.com.


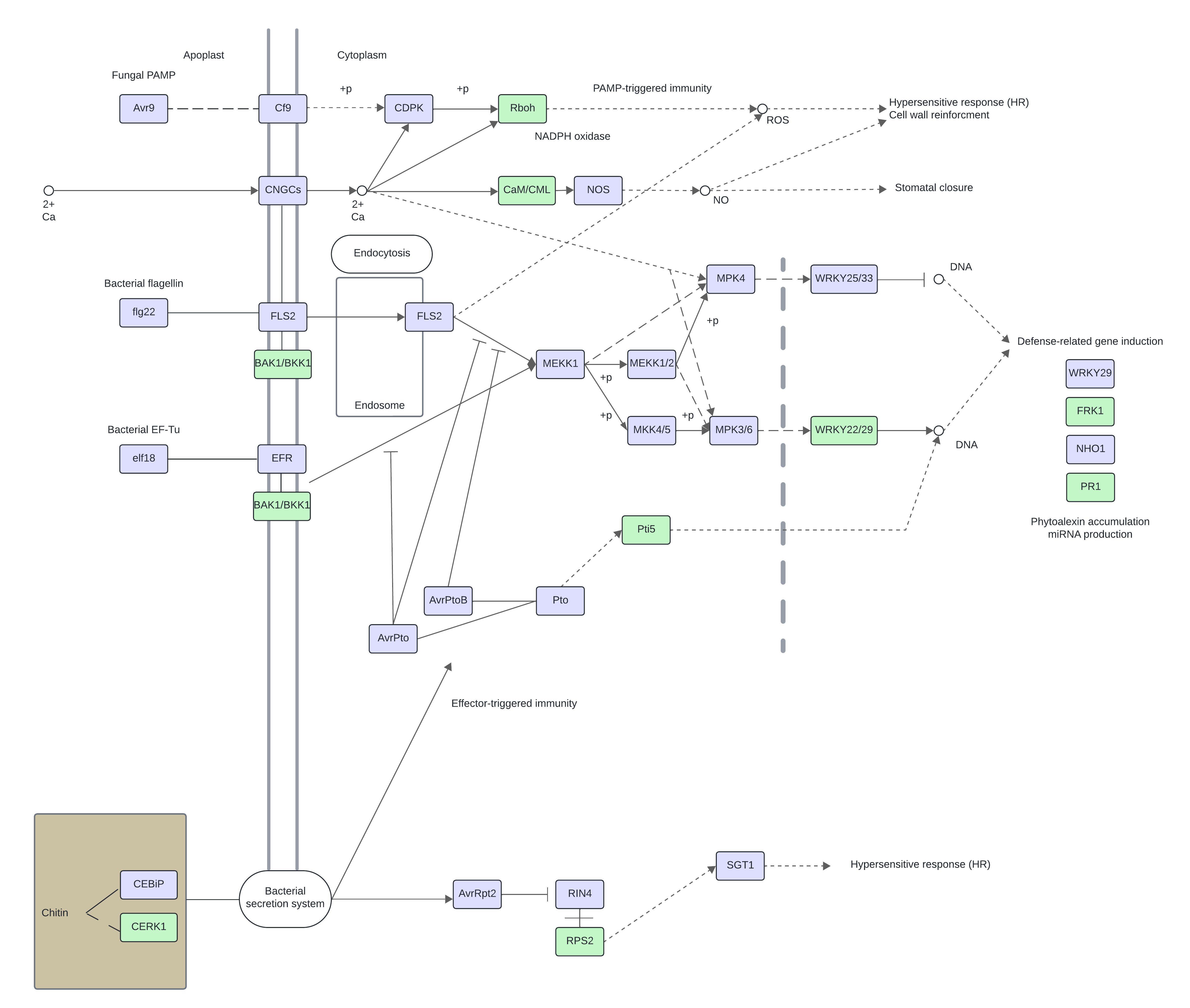


**Supplementary Figure 2.** KEGG map (ko04626: Plant–pathogen interaction) identified for upregulated genes in ripe mango fruit cv. Azúcar at 12 hpi. Genes *BAK1/BKK1, CERK1, CaM/CML, Rboh, WRKY22/29, FRK1, PR1, Pti5*, and *RPS2* are highlighted in green. Figure created with Lucidchart.com.


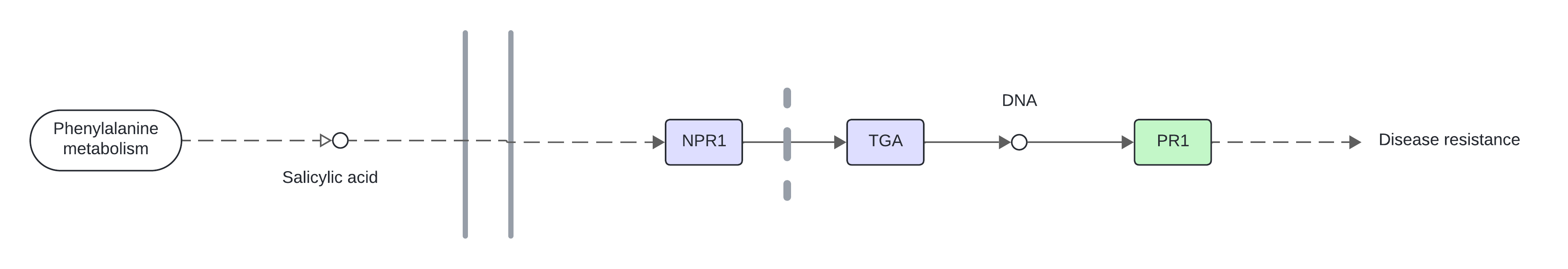


**Supplementary Figure 3.** KEGG map (k04075: Plant hormone signal transduction) identified for upregulated genes in ripe mango fruit cv. Azúcar at 12 hpi. The *PR1* gene is highlighted in green. Figure created with Lucidchart.com.


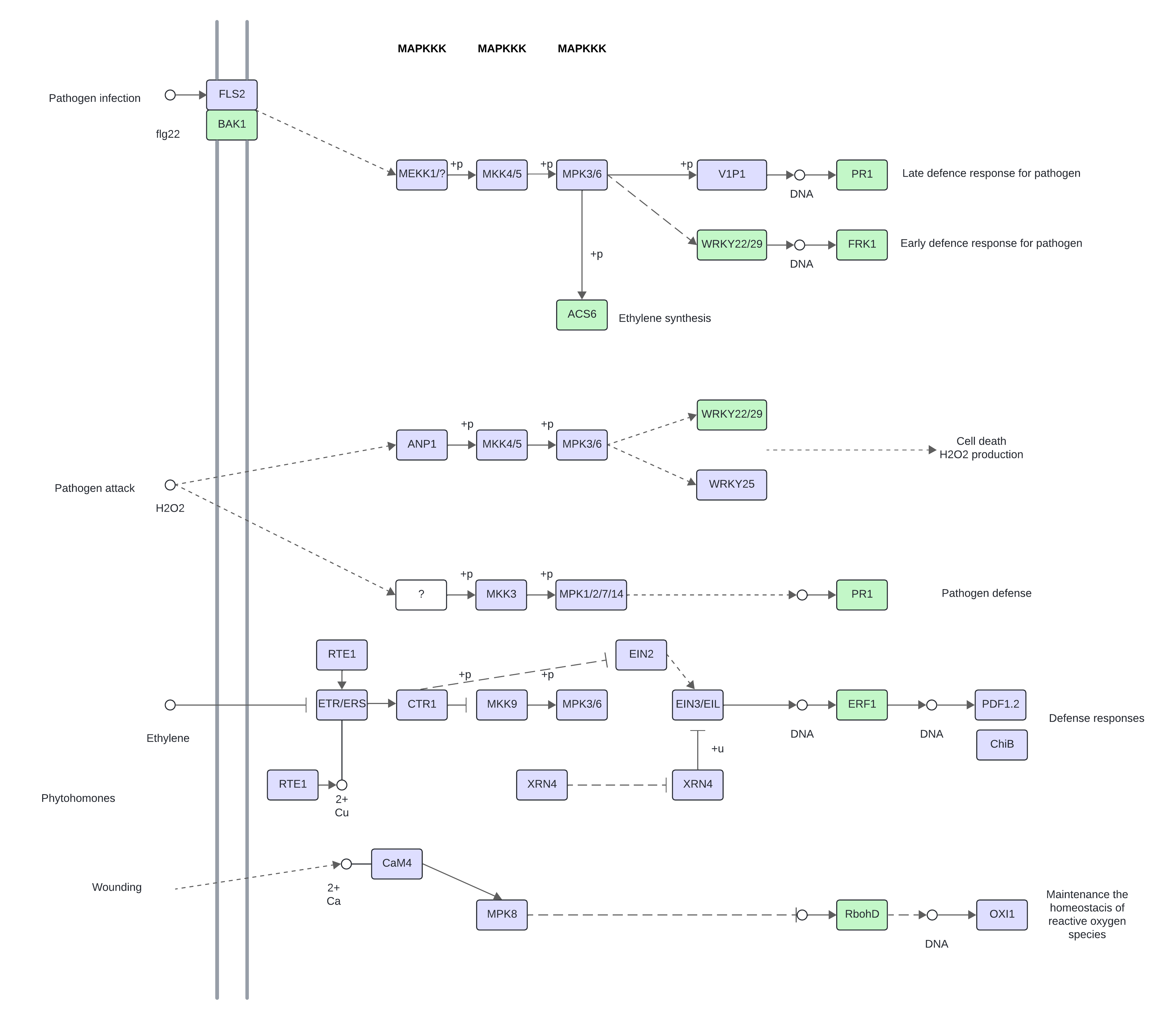


**Supplementary Figure 4.** KEGG map (k04016: MAPK signaling pathway – plant) identified for upregulated genes in ripe mango fruit cv. Azúcar at 12 hpi. Genes *BAK1, ACS6, WRKY22/29, PR1, FRK1, ERF1*, and *RbohD* are highlighted in green. Figure created with Lucidchart.com.
